# A Case of Hypofractionated Radiation Therapy for Early-Stage Breast Cancer in a Patient With Fabry Disease

**DOI:** 10.7759/cureus.54329

**Published:** 2024-02-16

**Authors:** Motoki Honda, Yojiro Ishikawa, Kengo Ito, Satoshi Teramura, Seki Yasuhiro, Takayuki Yamada

**Affiliations:** 1 Radiology, Tohoku Medical and Pharmaceutical University, Sendai, JPN; 2 Radiology, Tohoku University, Sendai, JPN

**Keywords:** fabry disease, intensity-modulated radiation therapy (imrt), hypofractionated rt, deep-inspiration breath-hold, breast cancer

## Abstract

Fabry disease is a metabolic disorder caused by a deficiency in lysosomal enzymes and is inherited as an X-chromosomal disorder. Patients with Fabry disease have a low incidence of cancer, and reports of malignant tumors, especially in the thoracic region, are rare. In this case report, we describe our experience with radiation therapy following breast-conserving surgery in a patient with left breast cancer and Fabry disease, and we review the existing literature. The patient, a woman in her 40s, required postoperative irradiation for left breast cancer (pT1N0M0). There were several patients with Fabry disease in her family, and the diagnosis of Fabry disease was made five years ago. Cardiac function evaluation revealed no significant abnormalities, but a myocardial biopsy had suggested the presence of Fabry disease. Due to the relatively preserved distance between the heart and the chest wall, the patient received heart-shielded three-dimensional conformal radiation therapy at a dose of 53.2 Gy in 20 fractions, without the use of deep-inspiration breath-hold or intensity-modulated radiotherapy. After treatment was completed, only mild radiation dermatitis was observed. Six months have passed since treatment, and there have been no serious adverse events.

## Introduction

Fabry disease is an X-linked inherited disorder in which globotriaosylceramide and related neutral sphingolipids accumulate due to decreased lysosomal alpha-galactosidase activity. Symptoms of Fabry disease include progressive damage to the brain, kidneys, and heart [1]. The annual incidence of this disease is 1 in 100,000 [2]. Fabry disease is an X-linked genetic disorder, but it is also known to occur in women, despite being an X-linked disorder [1]. Fabry disease is rarely associated with malignant tumors. The incidence rate ratio is 0.61, with a 95% confidence interval of 0.37 to 0.99 [3], and there are no clear guidelines for the treatment of breast cancer associated with Fabry disease due to limited reports of such cases. Radiation therapy (RT) for breast cancer, particularly left-sided breast cancer, has been shown to increase the risk of heart-related disease, with a reported 7.4% increased risk of heart disease per Gy for the average heart dose [4]. Therefore, it is necessary to minimize the radiation dose for patients with breast cancer complicated by Fabry disease, which can cause myocardial damage. In this case report, we present a case of postoperative irradiation for the left breast in a patient with Fabry disease.

## Case presentation

**Figure 1:**
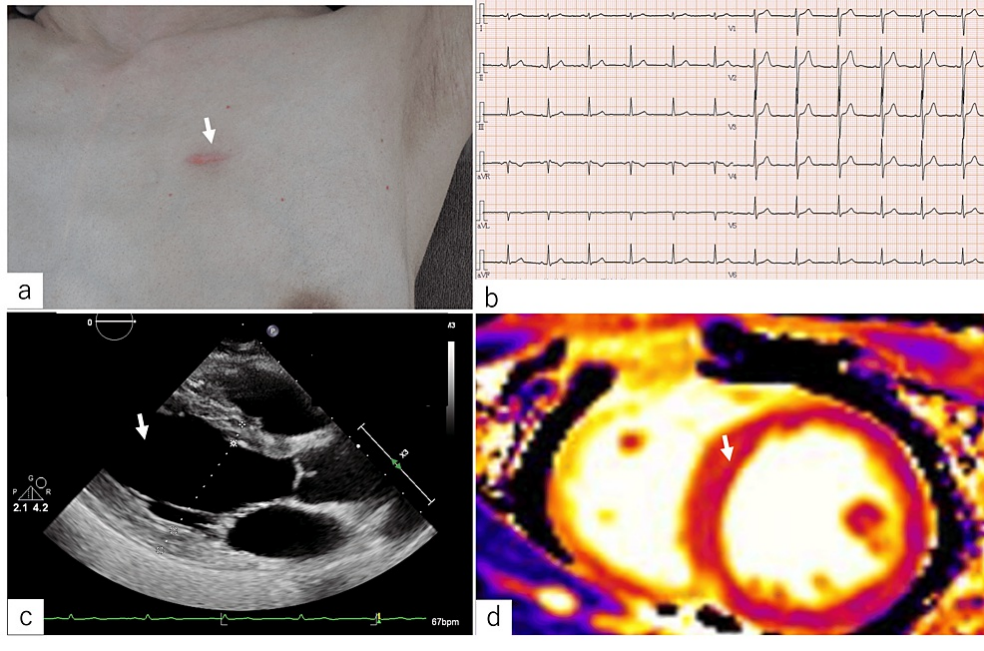
Postoperative gross findings and heart function examinations. Left chest wall: Postoperative subcutaneous mass excision in the upper inner quadrant of the left breast (a, arrow). Electrocardiogram findings: Sinus rhythm with no ST changes or obvious abnormalities (b). Echocardiography findings: Ejection fraction was 69% with no abnormal wall motion or ventricular enlargement (c, arrow). Magnetic resonance imaging: T1 mapping showed no native T1 value shortening or prolongation (d).

A Japanese woman in her 40s presented with a chief complaint of a painless subcutaneous mass in the left anterior thoracic region. She underwent a partial mastectomy, for a subcutaneous mass in the left anterior thoracic region at a local surgery hospital, and the postoperative pathological diagnosis was left breast cancer of invasive ductal carcinoma (IDC) (Figure 1a). The patient was referred to a general hospital for additional treatment, and imaging diagnosis and histopathology were reevaluated. Although the tumor size was 7 mm, IDC, scirrhous type, estrogen receptor (+), progesterone receptor (+), human epidermal growth factor receptor 2 (-), Ki-67: 20%, and the tumor was located in the upper inner quadrant of the left breast, there was no postoperative residual disease, lymph node metastasis, or distant metastasis, and the final diagnosis of left breast cancer (pT1cN0M0) was confirmed. While this was atypical of the usual breast-conserving surgery, it was determined that no additional surgery was necessary, and a plan was made to perform postoperative irradiation. Her medical history included Fabry disease and ulcerative colitis, both of which were asymptomatic at that time. An echocardiogram showed normal sinus rhythm with no obvious abnormalities (Figure 1b), and echocardiography showed a preserved ejection fraction of 69% and no regional wall motion abnormality (Figure 1c). A magnetic resonance imaging examination revealed no apparent abnormalities, and T1 mapping also showed no distinct abnormalities (Figure 1d).

**Figure 2:**
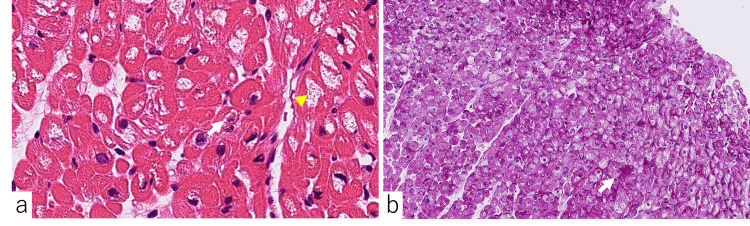
Myocardial biopsy performed five years earlier. (a) Hematoxylin and eosin staining was observed at 40x magnification, revealing cardiomyocyte size irregularities, vacuolar degeneration (arrowhead), and lipofuscin granule deposition (arrow). (b) Periodic acid-schiff (PAS) staining was observed at 20x magnification, indicating the presence of PAS-positive material in cardiomyocytes (arrow).

On the other hand, a myocardial biopsy performed five years earlier showed myocardial cell size irregularities, vacuolar degeneration, and lipofuscin granule deposition on hematoxylin and eosin staining (Figure 2a). Periodic acid-schiff (PAS) staining showed PAS-positive material in the myocardial cells, indicating degeneration of the myocardium (Figure 2b).

**Figure 3:**
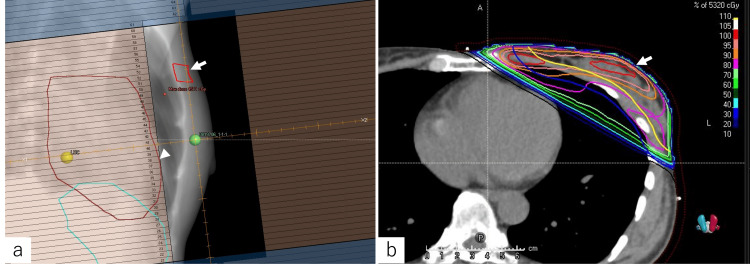
Radiotherapy plan. Beam's eye view of radiotherapy with three-dimensional conformal radiation therapy (3DCRT) shows tumor bed in the upper inner quadrant of the left breast (a, arrow), with the heart just barely shielded by a multileaf collimator (arrowhead). In the axial view of radiotherapy with 3DCRT, the heart exposure appears to be visually reduced due to the distance between the heart and chest wall (b, arrow).

Although no symptoms of Fabry disease were present, the effect of Fabry disease on the myocardium was evident, leading to the decision to consider treatment to minimize its effects on the myocardium. For RT, in addition to conventional three-dimensional conformal radiation therapy (3DCRT), deep-inspiration breath-hold and volumetric modulated arc therapy (VMAT) techniques were considered to minimize cardiac exposure. First, a conventional 3DCRT plan for 42.56 Gy/16 fractions was developed (Figures 3a, 3b).

**Figure 4:**
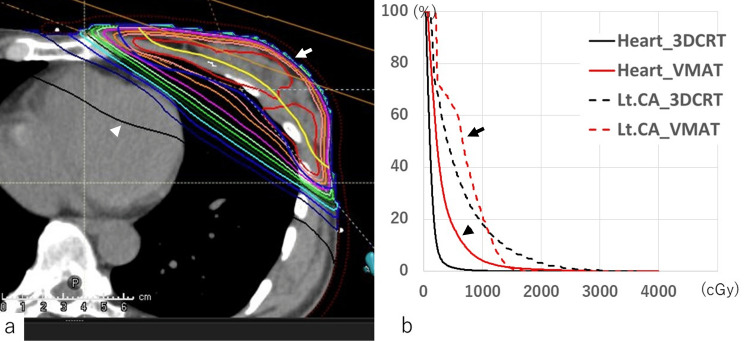
Volumetric modulated arc therapy simulation plan (a) The volumetric modulated arc therapy (VMAT) simulation plan was assessed as a means of minimizing cardiac exposure. The treatment planning software (RayStation) was utilized for a total dose of 42.56 Gy in 16 fractions, with consideration given to the following dose constraints for adjacent organs: Bilateral lung V20 < 10%, Dmean < 15 Gy; Heart V30 < 5%, Dmean < 5 Gy; left anterior descending coronary artery Dmean < 10 Gy, Dmax < 20 Gy; contralateral breast D1cc < 10 Gy. A dose distribution chart generated by VMAT indicates that a portion of the low-dose region extends over the heart (arrow). (b) In comparison to three-dimensional conformal radiation therapy (3DCRT), VMAT resulted in a larger low-dose area affecting the heart, as evidenced in the dose-volume histogram (DVH) for the left anterior descending branch of the coronary artery (CA) (arrowheads) and the heart itself (arrow).

Although deep-inspiration breath-hold (DIBH) irradiation was considered, it was decided not to use it because the distance from the heart to the chest wall under free breathing was adequately maintained in this case, as shown in Figure 3. Next, a VMAT simulation plan was created (Figures 4a, 4b) with dose constraints for surrounding organs for 42.56 Gy/16 fractions.

Treatment with VMAT was determined to be of no significant benefit to both the heart and coronary arteries because of the tendency for the low-dose area to be elevated. The final decision was to use 3DCRT for 42.56 Gy/16 fractions and an electron beam boost of 10.24 Gy/4 fractions with the heart shielded as much as possible by using a multileaf collimator. To avoid unscheduled cardiac exposure during irradiation, real-time scans were performed using optical projection and a camera system (Catalyst), and chest wall movement was monitored during irradiation. Although some erythema is evident, dermatitis is grade 1, and a simple chest X-ray taken six months after RT showed no evidence of pneumonia.

## Discussion

Fabry disease exhibits variations in the incidences of symptoms between classical and nonclassical subtypes and between men and women. Men with classical Fabry disease tend to experience clinical manifestations more frequently than do women with either phenotype. A previous study showed that the hazard ratio for the classical phenotype was significantly higher in men than in women (3.87, 95% confidence interval, 2.32 to 6.55; P<0.001) and that the hazard ratio for the nonclassical phenotype was also higher in men than in women (1.98, 95% confidence interval, 1.07 to 53.69; P<0.05 [5]. The actual incidence of Fabry disease in women may have been underestimated due to the lack of clinical symptoms. In a recent study, the incidence of Fabry disease was estimated to be approximately 1 in 7,683 [6]. Therefore, it is crucial to obtain comprehensive medical and family history information from the patient. Fabry disease is rarely reported, and to our knowledge, the only reported case of breast cancer treatment in a patient with Fabry disease was for a patient in Lebanon whose family was the only Fabry disease family in Lebanon. In that case, a left breast mass was treated with a total left mastectomy and axillary lymph node dissection followed by six cycles of chemotherapy (adriamycin 50 mg/m^2^ + cyclophosphamide 500 mg/m^2^ + 5-fluorouracil 500 mg/m^2^), local irradiation, and adjuvant hormone therapy. Although there is no detailed description of the irradiation [7], the emphasis was placed on minimizing cardiac effects. Our case involved hypofractionated RT for left breast cancer with Fabry disease, and no similar case has been reported to date. 

Whole breast irradiation (WBI) is the standard RT for breast-conserving surgery for early-stage breast cancer [8]. Recently, the results of hypofractionated WBI have been shown to be comparable to those of conventional fractionated WBI [9-11]. It has been reported that the incidences of acute myocardial infarction, ischemic heart disease, left coronary anterior descending stenosis, angina pectoris, pericarditis, and valvular heart disease are higher with left-sided irradiation than with right-sided irradiation [12]. Patients with Fabry disease are potentially at high risk for cardiac disease and cardiac dysfunction [1-3], and the cardiac risks associated with RT should be considered. However, there have been no reports of cardiac dysfunction in patients with Fabry disease undergoing RT. Although DIBH was not used in this case, previous reports have indicated that DIBH and VMAT can minimize cardiac dose parameters in left breast cancer patients undergoing postoperative RT. Fortunately, in this case, the distance between the heart and chest wall was adequate, allowing relatively safe irradiation using the conventional 3DCRT method. However, in cases in which the heart wall is in close proximity to the chest wall or the rib cage is deformed, such as in funnel chest (pectus excavatum) cases, there is a risk for high doses to the heart [13-15]. DIBH is an RT technique in which patients take a deep breath before treatment and hold their breath while radiation is administered, thus filling the lungs with air and separating the heart from the chest wall [16]. Although DIBH was not used in this case, previous reports have indicated that DIBH and VMAT can minimize cardiac dose parameters in left breast cancer patients undergoing postoperative RT [16,17]. Cardiac involvement in Fabry disease is a subtype characterized by the accumulation of glycolipids primarily in the heart muscle. Initially, thickening of the heart muscle and cardiac hypertrophy are observed, similar to hypertrophic cardiomyopathy. However, as the disease advances, partial or total left ventricular wall hypokinesia may develop, leading to dilated cardiomyopathy, heart failure, and arrhythmias [1-3]. In cases of cardiac complications as in our case, opting for total mastectomy instead of breast conservation is a viable option to avoid irradiation, although some patients may prefer not to undergo additional surgery [18,19].

There were three limitations identified in this case report. First, the short follow-up period of only six months was noted. Typically, radiation-induced heart damage manifests several years following RT. Minimizing radiation exposure to the heart is crucial for patients with heart conditions. Cardiac disease after irradiation can manifest over several years to more than 20 years [4], emphasizing the importance of ongoing careful monitoring of cardiac function. Secondly, the lack of case reports on RT in breast cancer patients with cardiac complications related to Fabry disease posed challenges in comparison with previous reports of radiation injury. Lastly, some reports suggested that patients with Fabry disease might have a higher incidence of cancer, contrary to the report that they were less likely to develop cancer [3,20]. It was difficult to provide a comprehensive discussion on the incidence of Fabry disease and cancer in this case report.

## Conclusions

We reported a case of left breast irradiation in a patient with Fabry disease, a condition that is challenging to diagnose and is often overlooked clinically. Given the scarcity of case reports on breast cancer complicated by Fabry disease and the asymptomatic nature of female Fabry disease patients, it is essential to pay attention not only to the patient's symptoms and medical history but also to the patient’s family history. There remain a limited number of reports on cancer development in Fabry disease, including breast cancer, and the accumulation of more cases is necessary to advance our understanding of RT for this condition.
